# Random X Inactivation and Extensive Mosaicism in Human Placenta Revealed by Analysis of Allele-Specific Gene Expression along the X Chromosome

**DOI:** 10.1371/journal.pone.0010947

**Published:** 2010-06-04

**Authors:** Joana Carvalho Moreira de Mello, Érica Sara Souza de Araújo, Raquel Stabellini, Ana Maria Fraga, Jorge Estefano Santana de Souza, Denilce R. Sumita, Anamaria A. Camargo, Lygia V. Pereira

**Affiliations:** 1 Laboratório de Genética Molecular do Departamento de Genética e Biologia Evolutiva, Universidade de São Paulo, São Paulo, Brazil; 2 São Paulo Branch, Ludwig Institute for Cancer Research, São Paulo, Brazil; 3 Genomic Engenharia Molecular, São Paulo, Brazil; Institut Curie, France

## Abstract

Imprinted inactivation of the paternal X chromosome in marsupials is the primordial mechanism of dosage compensation for X-linked genes between females and males in Therians. In Eutherian mammals, X chromosome inactivation (XCI) evolved into a random process in cells from the embryo proper, where either the maternal or paternal X can be inactivated. However, species like mouse and bovine maintained imprinted XCI exclusively in extraembryonic tissues. The existence of imprinted XCI in humans remains controversial, with studies based on the analyses of only one or two X-linked genes in different extraembryonic tissues. Here we readdress this issue in human term placenta by performing a robust analysis of allele-specific expression of 22 X-linked genes, including *XIST*, using 27 SNPs in transcribed regions. We show that XCI is random in human placenta, and that this organ is arranged in relatively large patches of cells with either maternal or paternal inactive X. In addition, this analysis indicated heterogeneous maintenance of gene silencing along the inactive X, which combined with the extensive mosaicism found in placenta, can explain the lack of agreement among previous studies. Our results illustrate the differences of XCI mechanism between humans and mice, and highlight the importance of addressing the issue of imprinted XCI in other species in order to understand the evolution of dosage compensation in placental mammals.

## Introduction

In mammals, dosage compensation of X-linked gene products between XX females and XY males is achieved by the transcriptional inactivation of all but one X chromosome per diploid cell in females early in embryogenesis. In marsupials, X chromosome inactivation (XCI) is imprinted, and the paternal X is inactivated in the embryo [1], [2]. Imprinted XCI is also found in Eutherians like mice, rats [3]–[5] and, although less extensively characterized, bovines [6], however exclusively in extraembryonic tissues. In cells of the embryo proper, either the paternal or the maternal X chromosome is inactivated in a random fashion.

Traditionally, the process of XCI has been best studied in the mouse, where it has been shown to be triggered by expression in *cis* of the noncoding *Xist* gene exclusively from the future inactive X (Xi), and to occur in two waves in the female pre-implantation embryo (reviewed in [7]). Imprinted XCI becomes evident as early as in the 4-cell stage [8]–[10], where expression of *Xist* exclusively from the paternal X (Xp) results in its inactivation. At the blastocyst stage, cells from the epiblast reactivate the paternal Xi , and then go through a second round of XCI, this time randomly choosing the paternal or the maternal X as the inactive one [9], [11].

Studies of XCI in human extraembryonic tissues date back to late 1970 s, when the most common X-linked marker used was glucose-6-phosphate dehydrogenase (*G6PD*) with its electrophoretic variant isoforms (Table 1). The analysis of G6PD in samples from term placentas provided conflicting results regarding the pattern of XCI in those tissues, where both random [12] and preferential inactivation [13]–[15] were reported. Contradicting conclusions were also obtained in studies analyzing other polymorphic X-linked loci in chorionic villi at different gestational ages (Table 1) [16]–[20].

**Table 1:** Review of the literature regarding XCI pattern in human extraembryonic tissues.

Material (origin)	Extraembryonic cell lineage		Locus	Results (*)	Authors' conclusion	Ref.
Placenta (full-term)	Placental membranes homogenate		*G6PD*	13 skewed (Xp inactive); 9 random	Preferential paternal inactivation	[13]
Placenta (full-term)	Chorionic villi		*G6PD*	9 random; 3 skewed (Xp inactive)	Random inactivation	[12]
Chorionic villi# (suction abortion)			*G6PD*	8 skewed in some degree (6 Xp inactive and 2 Xm inactive); 1 random	Random inactivation	[16]
Placenta (full-term)	Fresh amnion		*G6PD*	8 skewed (7 Xp inactive and 1 Xm inactive); 3 random	Preferential paternal inactivation	[14]
	Cultured amnion			22 skewed (16 Xp inactive and 6 Xm inactive); 10 random		
	Fresh chorion			11 random; 9 skewed (8 Xp inactive and 1 Xm inactive)		
	Cultured chorion			21 skewed (16 Xp inactive and 5 Xm inactive); 11 random		
	Cultured villi			16 skewed (15 Xp inactive and 1 Xm inactive); 10 random		
Placenta (full-term)	Cultured cytotrophoblasts isolated from several cotyledons		*G6PD*	7 skewed in some degree (5 Xp inactive and 2 Xm inactive); 5 completely skewed (Xp inactive)	Preferential paternal inactivation	[15]
Chorionic villi (elective abortion)	Fresh and cultured villi		*G6PD* and *HPRT*	4 random	Random inactivation	[17]
Chorionic villi# (diagnostic exam)	Fresh trophoblastic cells		*AR*	2 skewed (Xp inactive)	Preferential paternal inactivation	[18]
Placenta (full term)	2 adjacent fragments isolated from the same cotyledon		*AR*	5 skewed in some degree (2 Xp inactive; 2 Xm inactive; 1 either Xp or Xm); 2 completely skewed (1 Xp inactive and 1 either Xp or Xm); 1 random	Random inactivation	[27]
	9 non-adjacent fragments isolated from the same placenta			Heterogeneous pattern depending on the cotyledon		
Chorionic villi# (elective abortion)	Whole (fresh) branch villi		*PGK1*	5 skewed in some degree (always more Xp inactive); 1 random	Non-random inactivation	[19]
	Trophoblasts isolated from anchoring villi			19 skewed (13 Xp inactive and 6 Xm inactive); 11 random		
	Trophoblasts isolated from branch villi			17 skewed (14 Xp inactive and 3 Xm inactive); 21 random		
Chorionic villi# (diagnostic exam)			*FMR1*	2 random	Random inactivation	[33]
Chorionic villi#,¥ (elective abortion)	Cytotrophoblasts		*AR*	37 random; 17 skewed in some degree; 1 completely skewed	Both random and skewed inactivation	[20]
Human embryonic stem cells (H9 line)	Human ES cells at passages 45–48 differentiated in trophoblasts		*FMR1*	Non-random	Non-random inactivation	[30]

(#) villi from first trimester.

(¥) villi from second trimester.

(*) As scored by the *respective* authors. Xp, paternal X; Xm, maternal X.

Some factors may account for these controversies, including analysis of different tissues, small sample size and possible contamination with maternal DNA. Additionally, it is important to note that all those reports have relied on the analysis of only one or two X-linked loci in order to infer the activity of the entire chromosome (Table 1). However, some data indicate a possible variability in the expression status of some genes from the Xi among females [21], [22], and therefore, the analysis of a single locus may not be adequate to represent the expression activity of the whole X chromosome.

Here we take advantage of the vast number of human X-linked SNPs described [22], [23] to perform for the first time an analysis of allele-specific gene expression along the X chromosome in full-term placenta. Our data indicate a heterogeneous maintenance of the inactive state of genes on the Xi in that organ, confirming the importance of analyzing several X-linked loci in order to infer the pattern of XCI. Moreover, they show that the term placenta is composed of relatively large clonal populations with either the paternal or the maternal Xi, which could be interpreted as completely skewed XCI, and may explain the contradictory nature of the previous reports. As a consequence, we conclude that XCI is random in human placenta.

## Results

Samples were collected from the fetal portion of 22 full-term human placentas, and from the respective maternal oral mucosa cells. Each placenta sample was tested for maternal DNA contamination and to confirm maternal identity by PCR amplification of 17 microsatellites in different autosomes and the amelogenin locus (data not shown). In only one case (pl.05) the maternal DNA sample did not match with the placental specimen (data not shown), and therefore that maternal DNA was removed from the analysis.

**Figure 1:**
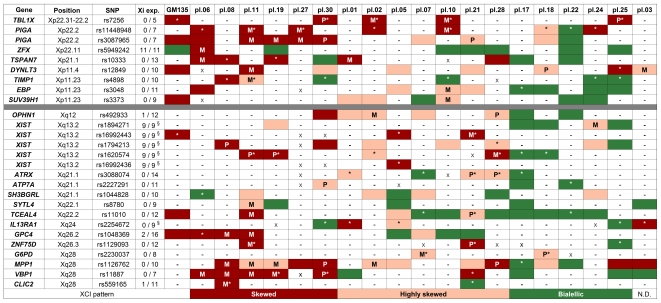
Summary of allele-specific X-linked gene expression in human placenta. Ratios of expressed alleles for each locus are shown: red (0∶100); pink (between 0∶100 and 20∶80); green (above 20∶80). Ratios of expressed alleles were scored by PeakPicker or visual analysis (*) of electropherograms. GM135, completed skewed XCI human fibroblast cell line. Placental samples (pl.) are grouped as showing predominantly completely skewed, skewed, or random inactivation. (ND) not determined. (-) non-informative locus; (X) informative locus; (M) expression from maternal allele; (P) expression from paternal allele. Column one: gene symbol; Column two: chromosomal position as in Vega Human View, v35 - Mar 2009 (http://vega.sanger.ac.uk/Homo_sapiens/index.html). Column three: SNP variant according to NCBI dbSNP BUILD129 (http://www.ncbi.nlm.nih.gov/SNP/). Column four: Gene expression on the Xi, where expression results are indicated as the number of primary human fibroblasts expressing each gene from the Xi per number fibroblasts tested, or (§) number of rodent/human somatic cell hybrids with the Xi that expressed the gene per number of hybrids tested [22].

We selected 27 SNPs in exons of 22 X-linked genes expressed in placenta whose transcriptional activity from the Xi had been previously analyzed in non-randomly inactivated primary human fibroblasts [22] (Figure 1, Table S1). Placental samples were genotyped for these X-linked SNPs, resulting in at least 5 informative SNPs per sample (average of 9 informative SNPs/sample). In addition, each SNP was informative in at least 4 samples (average of 10 informative samples/SNP) (Figure 1).

**Figure 2:**
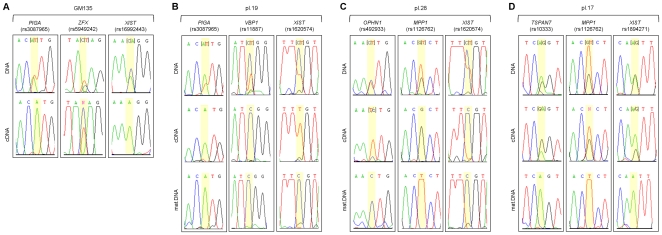
Analyses of expressed alleles in human term placenta. Examples of electropherograms of DNA and cDNA sequences of X-linked SNPs in (A) completely skewed fibroblast GM135; (B) pl.19; (C) pl.28; and (D) pl.17. Genes symbols and corresponding SNP ID are indicated above. Sequences from DNA and cDNA from cell line/placental samples, and corresponding maternal (mat.) DNA are shown. SNP position is highlighted in yellow. In (A) *ZFX* is shown as an example of a gene that escapes XCI.

To evaluate allele-specific gene expression, cDNA of RNAs from informative samples were genotyped by direct sequencing of the respective RT-PCR products. As a control we used a cell line of human fibroblasts with completely skewed XCI [24], [25], in which we were able to show monoallelic expression of 8 informative genes, including *XIST*, and biallelic expression of the escapee gene *ZFX* in accordance with the expression profile of the Xi [22] (Figure 2A).

**Figure 3:**
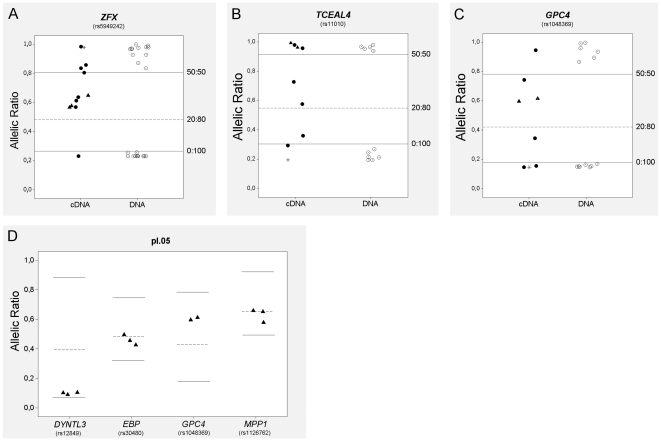
Quantification of ratio of expressed alleles using PeakPicker software. Solid lines indicate threshold levels for 0∶100 (lower) and 50∶50 (upper) ratios of expressed alleles. Dotted line indicates theoretical ratio of 20∶80. Open circles represent data from genomic DNA, filled circles from cDNA (filled triangles are experimental replicas), and asterisks from cDNA of completely skewed fibroblast GM135. Gene symbols and corresponding SNP ID are indicated. Analysis of (A) *ZFX* (escapes XCI), (B) *TCEAL4* and (C) *GPC4* (subjected to XCI) genes in different placentas; (D) Ratios of expressed alleles of different genes in pl.05 – threshold levels and theoretical ratio of 20∶80 is shown for each gene.

We applied this analysis to the collection of placental specimens, determining the origin of the expressed allele for each informative gene (Figure 1, Figure 2B–D). In contrast with results from the fibroblast cell line, where a clear mono or biallelic expression pattern was observed for each gene analyzed, many placental samples showed intermediate allelic ratios (Figure 2D) that were quantified with the PeakPicker software [26]. For each gene, DNA from heterozygous and homozygous samples were used to set up the threshold values of expected allelic ratios corresponding to random (50∶50) and completely skewed XCI (0∶100), respectively. Figure 3 shows representative examples of the PeakPicker analysis per gene and per sample. For the escapee gene *ZFX*, this analysis placed most samples in or close to the 50∶50 ratio of expressed alleles, reflecting its biallelic expression pattern (Figure 3A). In contrast, *TCEAL4* and *GPC4*, genes subjected to XCI, displayed a wider variability of allelic ratios among different samples, ranging from 0∶100 to 50∶50 (Figure 3B–C). Values of allelic ratios from experimental replicas fell within the same range, showing the robustness of the quantification method (Figure 3D). PeakPicker results were obtained for those electropherograms with phred scores greater than 20 (16 SNPs in 15 genes) (Figure S1, Figure S2). The remaining 11 SNPs were classified by visual analysis of the electropherograms (Figure 1).

Figure 1 summarizes our data after quantification. The ratios of expressed alleles observed in each locus were used to classify samples in three categories regarding the XCI pattern: completely skewed (allelic ratios at or below the 0∶100 threshold), skewed (allelic ratios between 0∶100 and 20∶80), and random (allelic ratios at or above 20∶80). We found consistency regarding the parental origin of the highest expressed allele within all samples classified as skewed or completely skewed, allowing us to score 9 samples as presenting preferential paternal XCI (pl.01, pl.02, pl.06, pl.07, pl.08, pl.10, pl.11, pl.19, and pl.27), and 3 with preferential inactivation of the maternal X (pl.21, pl.28, and pl.30). In addition, 5 of these 12 samples showed consistent opposite parental origin of the expressed *XIST* allele when compared to the other X-linked genes (Figure 1: pl.08, pl.11, pl.19, pl.21 and pl.28; Figure 2B, 2C). Five additional placental samples (Figure 1: pl.17, pl.18, pl.22, pl.24 and pl.25) presented patterns consistent with random XCI. A puzzling pattern was observed in pl.03, where two loci showed completely skewed pattern of inactivation, while two other loci presented random inactivation (Figure 1).

**Figure 4:**
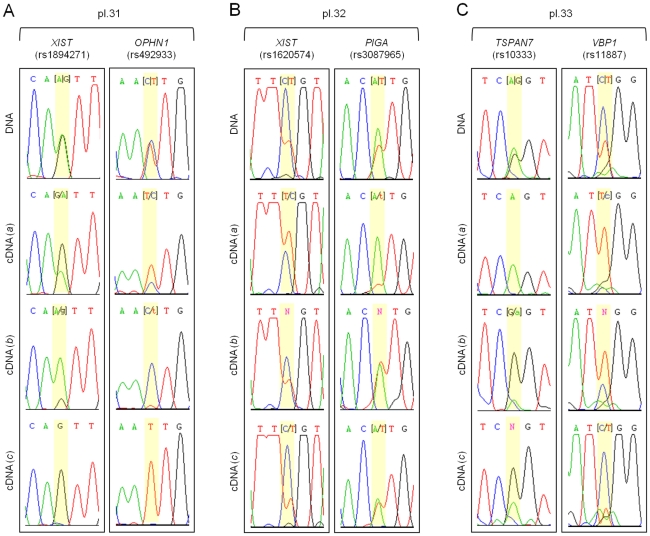
Mosaicism of the human full-term placenta regarding XCI. Electropherograms of DNA and cDNA sequences of X-linked SNPs in three different fragments of placentas (A) 31, (B) 32 and (C) 33. Gene symbols and corresponding SNP ID are indicated. SNPs are highlighted in yellow.

To reconcile the variable patterns observed, we proposed that XCI is in fact random in placenta, which is organized in patches of cells with either the maternal or the paternal inactive X, as previously observed by analysis of the *AR* locus [27]. To test this hypothesis with more X-linked genes, we collected 3 additional placentas and analyzed allele-specific gene expression in three different non-adjacent fragments of each (Figure 4). While allele-specific gene expression analysis of *XIST* and *OPHN1* in fragment *(a)* from placenta 31 indicated random XCI pattern, fragments *(b)* and *(c)* from the same placenta showed skewed and completely skewed XCI, respectively (Figure 4A). Similar results were observed for *XIST* and *PIGA* genes in placenta 32, and *TSPAN7* and *VBP1* in placenta 33 (Figure 4B, 4C). Together, these data corroborate our hypothesis and demonstrate that each isolated fragment from a single placenta may yield different results regarding patterns of XCI.

## Discussion

Imprinted inactivation of the paternal X chromosome occurs in marsupials as the ancestral mechanism of dosage compensation between the genders in Therians (reviewed in [7]). During the 147 million years that separate Metatheria from Eutheria [28], this process acquired an additional pathway exclusively in the epiblast cells, consisting of reactivation of the Xi and a second round of XCI, this time random, as observed in mice [9], [11]. The question remains: how much more has XCI evolved from that mammal to humans?

XCI in human embryos has been recently shown to be present as early as at the 8-cell stage [29], indicating that pre-implantation XCI has been evolutionary conserved between humans and mice. However, it has not been determined whether at that stage inactivation was imprinted or random. Although imprinted XCI in humans has been the subject of several studies for the last 30 years, a careful analysis of published results and their conclusions reveals that this issue is still controversial (reviewed in [7], [29]). Even the two most recent articles on this subject have reported random and non-random inactivation in different extraembryonic tissues [20], [30]. Since all those studies relied on data from only one or two X-linked loci (Table 1), we set forth to readdress this issue in human term placenta, performing a more robust analysis of allele-specific gene expression along the X chromosome. This allowed us to conclude that XCI is random in that organ.

The analysis of multiple X-linked loci in each sample revealed that the expression behavior of the chromosome as a whole may not be inferred from that of a single locus. For instance, analysis of *TIMP1* alone would indicate skewed and random XCI in placentas pl.11 and pl.30, respectively (Figure 1). However, considering the data from the other informative loci, those samples are better classified as presenting completely skewed XCI. In fact, although the data on allele-specific expression was consistent within each sample in terms of parental origin of the highest expressed allele, in most samples the allelic ratios varied among different loci (Figure 1), suggesting a heterogeneous relaxation of the epigenetic state along the Xi in this organ. Indeed, Xi chromosomes from term placenta have been shown to be more amenable to reactivation in human/rodent somatic cell hybrids than those from other somatic tissues [31], [32]. Our data in the *in vivo* system is compatible with the reactivation of some genes on the Xi, and thus corroborate those observations *in vitro*. It is interesting to notice that such extensive variation was not observed in the GM135 cell line, nor in a panel of 30 primary human fibroblasts [22], which indicates that maintenance of XCI may indeed be less stringent in placenta. A similar analysis in different human tissues will be important to show whether the relaxation of XCI is restricted to extraembryonic tissues, or it is a more general feature of this epigenetic control. In summary, our data show that the expression behavior of the Xi in human placenta can be heterogeneous, and justify the analysis of multiple loci in order to infer the pattern of XCI. Thus, we argue that a similar multi-loci approach should be employed in order to confirm imprinted XCI in bovine placenta, which has been determined by the analysis of a single X-linked gene [6].

Finally, the analysis of a considerable number of samples allowed us to identify diverse patterns of XCI only consistent with random inactivation in the placenta, and the arrangement of this organ in patches of cells as large as 8 mm^3^ (see Materials and Methods) with the same Xi. The analysis of different non-adjacent fragments of the same specimen confirmed this mosaicism, and is in accordance with results obtained independently for the *AR* and the *FMR1* genes in term placenta and chorionic villi, respectively [27], [33]. Actually, if one considered the extensive mosaicism of those tissues, most of the data in the literature would be in agreement with random XCI in the human extraembryonic lineage, although some of the authors do not conclude that (Table 1). The only two studies that identified exclusively non-random XCI had important limitations: the first one relied on data from only two samples [18]; while the second was performed in trophoblast cells derived from the H9 line of human embryonic stem cells [30], which has recently been shown to present instability of the epigenetic state of the X chromosome [34], [35], and may not be modeling human XCI adequately *in vitro*.

Most of the currently known molecular mechanisms involved in XCI are conserved between humans and mice (reviewed in [7]). However, there are fundamental differences in that process between the two species, including the structure of the *TSIX/Tsix* gene (reviewed in [36]), and lack of imprinted XCI in extraembryonic tissues as thoroughly characterized here. It is interesting to notice that several autosomal genes imprinted exclusively in the murine placenta are not under this epigenetic control in humans [37]. Since the epigenetic marks of imprinted autosomes and of the imprinted Xi are similar in mouse placenta (reviewed in [38], [39]), one may envision that imprinted XCI in human extraembryonic tissues was lost during evolution in conjunction with autosomal imprinting. Nevertheless, it is noteworthy the preponderance of placental samples with preferential expression of maternal alleles, indicating that, although imprinted XCI was lost during evolution, a proliferative advantage may remain for cells that inactivate the Xp in human placenta. In light of our data, it will be important to address the issue of imprinted XCI in other Eutherians in order to understand the evolution of dosage compensation in placental mammals.

## Materials and Methods

### Samples

Twenty two human full-term placentas with corresponding maternal oral mucosa samples were collected at Amparo Maternal Obstetric Clinic (São Paulo, Brazil), with parental fully informed consent and approval by the local Institutional Ethics Committee. Only placentas resulting from normal pregnancies and delivery of a healthy female child were included in this study. Placental fragments were collected from the fetal portion near the umbilical cord insertion, washed several times in PBS to remove traces of maternal blood, and rapidly submerged in RNA stabilizing solution (RNA*later*™ QIAGEN) (samples 1–30). For placentas numbered 31, 32 and 33, three fragments were obtained from distinct nonadjacent regions of the same placenta and individually processed in a similar way. Maternal oral mucosa samples were stabilized in 50 mM NaOH.

### Nucleic acid extraction

Maternal genomic DNA was extracted as described [40]. Placental genomic DNA was extracted from 25 mg fragments, previously digested with 360 µL of lysis buffer (Amersham Biosciences, GE) and 40 µL of proteinase K (20 mg/mL) at 55°C over night, using GFX™ Genomic Blood DNA Purification Kit according to manufacturer's protocol (Amersham Biosciences, GE).

Total RNA was prepared from up to 100 mg (4–8 mm^3^ fragments) of placental tissue using Trizol®Reagent (Invitrogen) according to manufacturer's protocol, previous digested with 20 mg/ml proteinase K for 30 minutes at 55°C. To avoid DNA contamination, rigorous DNase treatment with Turbo DNA-Free (Ambion) was performed, following manufacturer's instructions. One to 2 µg of total DNase-treated RNA were reverse transcribed using M-MLV (Invitrogen) according to manufacturer's protocol. To test for DNA contamination, cDNA synthesis was also performed in the absence of reverse transcriptase (minus-RT control).

### SNP selection and primers design

Based on the human X chromosome sequence [23], NCBI dbSNP BUILD 129 (http://www.ncbi.nlm.nih.gov/SNP), and the expression profile on the Xi in a panel of 30 human primary fibroblasts [22], we selected 27 SNPs located in coding regions of 22 X-linked genes expressed in placenta. All primers were designed avoiding annealing in known SNPs regions. The list of SNPs and primers is presented in Table S1.

### Genotyping and analysis of allele-specific expression

Fifty to 100 ng of DNA or cDNA were used as templates for PCR amplification of the region surrounding each SNP with the primers listed in Table S1. PCR conditions are available on request. Before sequencing, PCR products were separated in 6% poliacrilamyde gel electrophoresis, and visualized by silver staining to exclude assays showing any amplification from the minus-RT control reaction and from the minus-template PCR control. Sequencing was carried out using those same primers and the BigDye® Terminator v3.1 Cycle Sequencing Kit (Applied Biosystems). Sequencing products were separated on an ABI Prism® 3100 Genetic Analyzer, following manufacturer's instructions (Applied Biosystems). Most samples were analyzed at least twice for each SNP, including distinct cDNA synthesis, RT-PCRs and sequencing assays.

### Quantification of allele-specific gene expression

Allelic expression levels were determined using the PeakPicker software specifically developed for relative quantification of peaks in sequencing electropherograms [26]. Because peak heights vary depending on sample, base type and their position within the sequence, the PeakPicker software carries out a normalization step in which the SNP allele height is compared to the height of reference peaks in flanking sequence. Default normalization settings were applied to quantify the relative amount of the two alleles measured from the electropherogram based on peak intensity of the two polymorphic bases. Subsets of informative heterozygotes, at least five for each SNP, were identified and their cDNA was amplified in identical conditions to verify the peak height ratio between bases corresponding to the SNP. We limited our PeakPicker analysis to sequence traces in which 70% of the bases within a 21 base window flanking the SNP presented phred quality score >20 [41], [42]. Ratio values above 1 were transformed to 1/(ratio) to set all of them in a 0–1 scale and then adjusted to the mean of the peak intensity ratios from DNA samples. Genomic DNA from heterozygous and homozygous samples were used to set up a threshold for allelic ratios of 50∶50 and 0∶100, respectively, for each gene. The normalized heterozygote and homozygote ratios of genomic DNA samples were then used to estimate the methodological variability and establish a 99% confidence interval (CI) for 50∶50 and 0∶100 ratios of expressed alleles, respectively. The 99% CI was calculated assuming that normalized peak height ratios of DNA samples are normally distributed according to the Anderson-Darling test. In addition, a theoretical threshold of 20∶80 ratio was calculated for each gene by dividing the respective interval between 50∶50 and 0∶100. The pattern of XCI for each sample was based on the analysis of allelic ratios of all informative loci. Allelic ratios above 20∶80 were indicative of random XCI [43]; between 20∶80 and 0∶100 were considered as skewed XCI; and only those ratios at or below 0∶100 were classified as completely skewed XCI.

## Supporting Information

Table S1 Genes analyzed and respective PCR primers used.
(0.03 MB XLS)

Figure S1 Quantification of ratio of expressed alleles per gene using PeakPicker software. Solid lines indicate threshold levels for 0:100 (lower) and 50:50 (upper) ratios of expressed alleles. Dotted line indicates theoretical ratio of 20:80. Open circles represent data from genomic DNA, filled circles from cDNA (filled triangles are experimental replicas), and asterisks from cDNA of completely skewed fibroblast GM135. Gene symbols and corresponding SNP ID are indicated.
(2.26 MB TIF)

Figure S2 Quantification of ratio of expressed alleles per sample using PeakPicker software. PeakPicker results for all informative SNPs in each placental (pl.) sample are shown. For each gene, solid line indicates threshold levels for 0:100 ratio of expressed alleles, and dotted line indicates theoretical ratio of 20:80. Filled circles represent data from cDNA. Gene symbols are indicated.
(4.88 MB TIF)
