# Supplementary figures and images for: Random X Inactivation and Extensive Mosaicism in Human Placenta Revealed by Analysis of Allele-Specific Gene Expression along the X Chromosome
Source: PLoS One. 2010 Jun 4;5(6):e10947. doi: 10.1371/journal.pone.0010947 (PMC2881032; doi:10.1371/journal.pone.0010947)

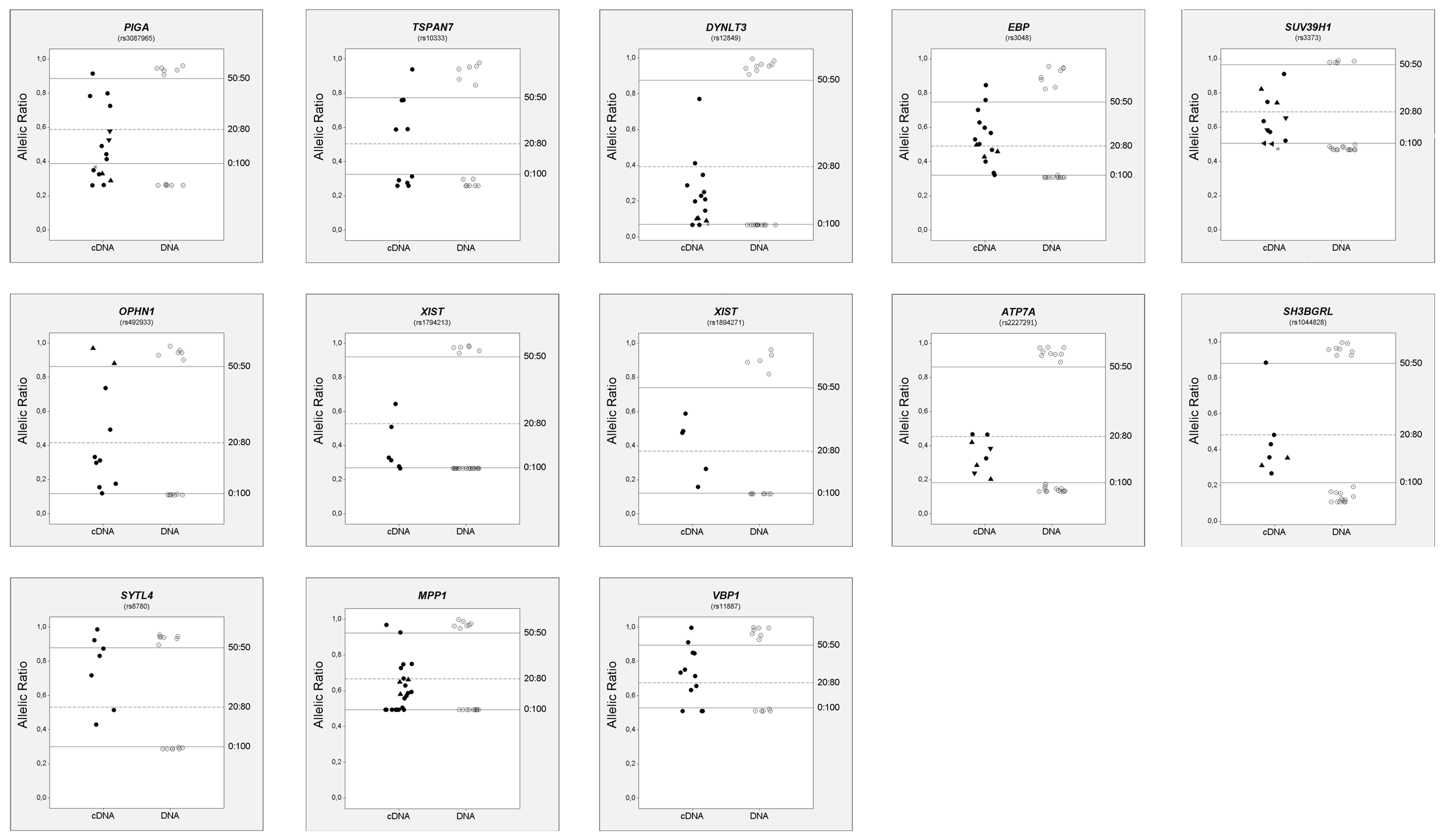

Supplement: Figure S1 — Quantification of ratio of expressed alleles per gene using PeakPicker software. Solid lines indicate threshold levels for 0:100 (lower) and 50:50 (upper) ratios of expressed alleles. Dotted line indicates theoretical ratio of 20:80. Open circles represent data from genomic DNA, filled circles from cDNA (filled triangles are experimental replicas), and asterisks from cDNA of completely skewed fibroblast GM135. Gene symbols and corresponding SNP ID are indicated. (2.26 MB TIF) [file pone.0010947.s002.tif]

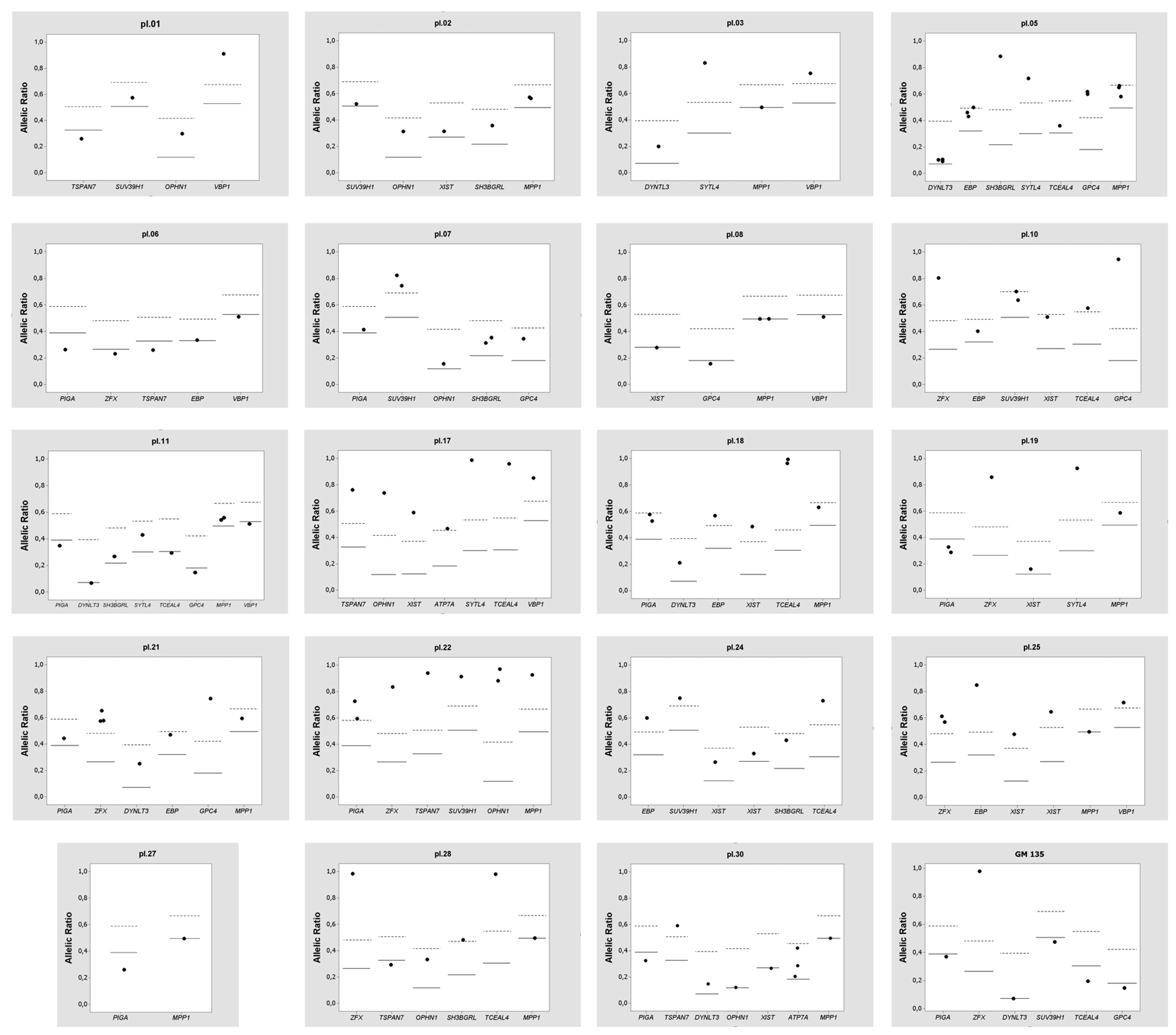

Supplement: Figure S2 — Quantification of ratio of expressed alleles per sample using PeakPicker software. PeakPicker results for all informative SNPs in each placental (pl.) sample are shown. For each gene, solid line indicates threshold levels for 0:100 ratio of expressed alleles, and dotted line indicates theoretical ratio of 20:80. Filled circles represent data from cDNA. Gene symbols are indicated. (4.88 MB TIF) [file pone.0010947.s003.tif]
